# Aging disrupts locus coeruleus‐driven norepinephrine transmission in the prefrontal cortex: Implications for cognitive and motor decline

**DOI:** 10.1111/acel.14342

**Published:** 2024-09-23

**Authors:** Evgeny Budygin, Valentina Grinevich, Zhong‐Min Wang, María Laura Messi, William Ryan Meeker, Jie Zhang, William Matthew Stewart, Carol Milligan, Osvaldo Delbono

**Affiliations:** ^1^ Department of Internal Medicine, Sections on Gerontology and Geriatric Medicine Wake Forest University School of Medicine Winston‐Salem North Carolina USA; ^2^ Department of Obstetrics and Gynecology Wake Forest University School of Medicine Winston‐Salem North Carolina USA; ^3^ Department of Translational Neuroscience Wake Forest University School of Medicine Winston‐Salem North Carolina USA

**Keywords:** aging, Gait, locus coeruleus, memory, mobility, noradrenergic neurons, prefrontal cortex

## Abstract

The locus coeruleus (LC)‐prefrontal cortex (PFC) circuitry is crucial for cognition, planning, posture and mobility. This study examines the role of norepinephrine (NE) in elucidating the neurobiological basis of age‐related cognitive and motor declines. Aged mice exhibited reduced spatial learning, impaired memory, decreased physical endurance, and notable changes in locomotor behavior. The neurochemical foundations of these deficits were investigated through fast‐scan cyclic voltammetry to measure NE release in the PFC and LC, both in vivo and in brain slices. Additionally, oxygen levels were monitored as a proxy for PFC neuronal function, and NE levels were analyzed in the extracellular space via microdialysis and total content in the PFC. Aged mice exhibited a frequency‐dependent increase in NE release in the PFC upon LC stimulation, suggesting alterations in neural responsiveness due to aging. We also recorded slower NE reuptake rates and increased NE content and neuronal activity, indicated by higher oxygen levels and facilitated neuron activation due to membrane depolarization recorded via whole‐cell patch‐clamp. To understand the basis for LC‐driven NE surges in the PFC with aging, we examined the expression levels of two proteins critical for presynaptic NE release and NE reuptake: the α2a‐adrenergic receptor and the NE transporter. Both showed a significant decrease in the PFC with aging. These findings support the concept that aging significantly alters the structural and functional dynamics within the LC‐PFC neural circuit, impacting NE modulation and neuronal activity, which may underlie the observed declines in cognitive and motor functions in aging populations.

## Graphical abstract

This study reveals age‐related changes in the locus coeruleus (LC)‐prefrontal cortex (PFC) circuit, including facilitated LC neuron activation, altered norepinephrine (NE) dynamics with increased NE content, and heightened neuronal activity, indicated by elevated oxygen levels in the PFC. Significant decreases in the expression of the α2a‐adrenergic receptor and norepinephrine transporter (NET) suggest NE transmission alterations. These findings imply that aging affects both the NE regulation and electrical properties of the LC‐PFC circuit, potentially contributing to cognitive and physical performance declines.

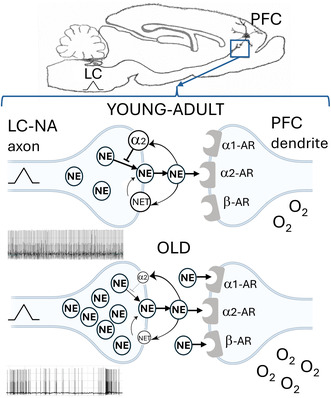

**Abbreviations**
A: ampere
aCSF: artificial cerebrospinal fluid
ANOVA: analysis of variance
AP: anterior‐posterior
BCA: bovine calf serum
CA: catecholamine
Dbh: dopamine beta hydroxylase
DMSO: dimethylsulfoxide
DV: dorsal‐ventral
EDTA: ethylenediaminetetraacetic acid
ECL: enhanced chemiluminescence
FSCV: fast‐scan cyclic voltammetry
fMRI: functional magnetic resonance imaging
HPLC‐ECD: high‐performance liquid chromatography‐electro‐chemical detector
Hz: hertz
I.P: intraperitoneal
IR‐DIC: infrared differential interference contrast
KI: knockin
LC: locus coeruleus
ML: medial‐lateral
Min: minute
MRB: methylreboxetine
NE: norepinephrine
NET: norepinephrine transporter
NFDMS: non‐fat dry milk
Osm: osmol
PFC: prefrontal cortex
PIC: protein inhibitor cocktail
PVDF: polyvinylidene fluoride or polyvinylidene difluoride
RIPA: radioimmunoprecipitation assay buffer
SD: standard deviation
SEM: standard error of the mean
TBST: tris‐buffered saline with tween 20
V: volt
2D: two‐dimensional

## 1 INTRODUCTION

Cognitive function has been linked with the onset of mobility impairments in community‐dwelling elders, (Buchman et al., 2011) and slower gait is an indicator of diminished cognitive function, (Rosano et al., 2004) as well as a strong predictor of dementia. (Verghese et al., 2007) As individuals age, gait tends to decline at a faster pace than cognition. Furthermore, individuals with motor decline are often subject to a more rapid deterioration in visuospatial, executive, and language functions, as evidenced by longitudinal studies. (Jayakody et al., 2021; Montero‐Odasso et al., 2018) We have recently demonstrated that accelerated sarcopenia precedes learning and memory impairments in a mouse model of tauopathy and Alzheimer's disease.(Longo et al., 2024) Given these associations, it is imperative to examine the primary central nervous system regions responsible for planning complex cognitive behaviors and mobility, as well as the dynamics of their connectivity. Such investigations are essential for understanding how these associations deteriorate with age. Identifying modifiable risk factors for the so‐called motoric cognitive risk syndrome could improve the detection of high‐risk individuals and assist in developing interventions to prevent cognitive decline during aging.

Despite the established link between cognitive decline and motor impairments in aging populations, the specific neural mechanisms underlying these associations remain poorly understood. The role of the locus coeruleus (LC) and its projections to the prefrontal cortex (PFC) in mediating these processes, particularly through norepinephrine (NE) modulation, has not been fully elucidated. The integrated function of the LC‐PFC circuitry in coordinating cognition and motor control, particularly in the context of aging, represents a significant gap in our understanding. Moreover, how age‐related changes in NE dynamics, such as altered release and reuptake, contribute to the observed declines in both cognitive and motor functions remains unclear.

The LC, the brain's primary source of NE, (Aston‐Jones & Cohen, 2005) interconnects with other neuron clusters in the brainstem to form a central hub that intersects cognition, locomotion, and skeletal muscle regulation.(Delbono et al., 2022) These clusters extend projections to various regions of the central nervous system, including local segmental areas in the brainstem, cephalic areas such as the telencephalon and diencephalon, and caudal regions like the ventral, lateral, and dorsal horns of the spinal cord. (Bruinstroop et al., 2012; Delbono et al., 2022) The role of noradrenergic neurons in higher brain function has been studied in the contexts of health and disease, (Chandler et al., 2019; Mulvey et al., 2018) while recent research has begun to explore its involvement in controlling neuromuscular transmission and skeletal muscle innervation and mass, which are vital components of locomotion. (Khan et al., 2016; Rodrigues et al., 2018; Rodrigues et al., 2021; Rodrigues et al., 2020; Straka et al., 2021) Given its ability to integrate both ascending and local information, as well as its projections to areas like the PFC, the LC serves as a critical relay point for investigating age‐dependent decline in motor coordination and cognition.

The PFC forms connections with the LC, (Valentino et al., 2017) which are vital for memory, cognition, planning, postural control, and locomotion. (Zwergal et al., 2012) This connectivity also contributes to the somato‐cognitive action network that integrates motor and autonomic body control with action planning. (Gordon et al., 2023) The pharmacological inhibition of presynaptic α2‐adrenergic autoreceptors in the PFC, which form part of the NE exocytosis inhibitor loop, significantly increases motor activity.(Ma et al., 2005) This underscores the vital role of NE projections from the LC to the PFC in regulating locomotor activity. Additionally, the interplay between the PFC and the LC's noradrenergic system is essential in regulating adaptive behavior in dynamic environments, particularly by managing inhibitory control in older adults.(Tomassini et al., 2022).

This study is designed to address this critical gap in knowledge by employing a comprehensive, multi‐modal approach to examine the LC, PFC, and LC‐PFC circuitry. It combines behavioral assessments, neurochemical measurements, and electrophysiological recordings. Unlike previous studies, this research simultaneously investigates the LC‐PFC circuitry's role in both cognitive and motor declines during aging. By utilizing fast‐scan cyclic voltammetry, microdialysis, and whole‐cell patch‐clamp techniques, the study provides real‐time insights into NE release dynamics and neuronal activity in both in vivo and ex vivo settings. Additionally, the examination of α2a‐adrenergic receptor and NE transporter expression levels offers a molecular perspective on the mechanisms underlying NE modulation with aging. (Benarroch, 2018) This integrative approach enables a more detailed understanding of how the LC‐PFC circuit coordinates cognitive and motor functions and how its disruption leads to age‐related declines, thereby offering novel insights that could inform therapeutic strategies to mitigate these effects in aging populations.

## 2 METHODS

### 2.1 Animals

Young adult (3–5 months) and old (21–23 months) C57BL/6 mice and young adult congenic B6.Cg‐Dbh^tm3.2(cre)Pjen^/J (Tillage et al., 2020) also known as Dbh^cre^ KI (Lee et al., 2024) (The Jackson Laboratory, strain 033951) were used. An equal number of males and females were included in all experiments. As there were no significant differences in outcome values between the sexes, their data were pooled together. Mice were maintained in a pathogen‐free area of the Wake Forest University School of Medicine (WFUSOM) Animal Research Program at 21°C and a 12:12 h dark/light cycle until the day of the experiment. The mice had ad libitum access to standard chow and continuous access to drinking water throughout the study. All experimental procedures were conducted in compliance with the National Institutes of Health Laboratory Animal Care Guidelines. The WFUSOM Institutional Animal Care and Use Committee approved Protocol A21‐172 for this study.

### 2.2 Fast‐scan cyclic voltammetry in vivo recording

Electrically‐evoked catecholamine release was recorded in the PFC of anesthetized mice. The mice were anesthetized with a single intraperitoneal injection of urethane (1.5 g/kg), and positioned in a stereotaxic frame. Drilled openings allowed for the insertion of electrodes into the brain. A stimulating electrode (Plastics One, Roanoke, VA, USA) was inserted into the LC (AP: −5.3 mm, ML: 0.75 mm DV: −3.2 mm), while a carbon fiber working microelectrode was placed in the ipsilateral PFC (AP: 1.8 mm, ML: 0.25 mm, DV: −2.0 mm) (Figure S1), and an Ag/AgCl reference electrode was implanted into the brain tissue of the contralateral hemisphere. The electrodes were connected to a voltammetric amplifier interfaced with a computer running specialized software (Tar Heel CV 4.8, Chapel Hill, NC, USA). Evoked catecholamine release was detected at the carbon fiber electrode every 100 ms by applying a triangular waveform (−0.4 V to +1.3 V and back to −0.4 V vs. Ag/AgCl) at a rate of 400 V/s and a frequency of 10 Hz, as previously described. (Deal et al., 2020) The signal was validated by a background‐subtracted cyclic voltammogram characterized by oxidation and reduction peaks occurring at ~ + 0.65 and − 0.2 V, respectively.

For pharmacological verification of the measured catecholamine, once a stable baseline signal was acquired (3 signals with amplitudes within 10% variability), raclopride (a dopamine D2 receptor antagonist, 2 mg/kg, i.p.; Tocris, Minneapolis, MN), idazoxan (an adrenergic alpha‐2 receptor antagonist, 5 mg/kg, i.p.; Millipore‐Sigma, Burlington, MA), or saline was injected (Figure S2). Electrically‐evoked (60 Hz, 60 pulses, 300 μA) catecholamine effluxes following a compound injection were measured at 10‐min intervals for 60 min. To explore the difference in frequency‐dependence of NE release between adult and old mice, 1‐s electrical stimulations (300 μA) of the LC were made at different frequencies (5, 10, 20, 30, 40, 50, and 60 Hz) every 10 min.

The depletion protocol, adopted from a previous study, (Nemets et al., 2022) included three consecutive stimulations (60 Hz, 600 pulses, 300 μA), applied at 1–2 s intervals. Then, regular stimulation (60 Hz, 60 pulses) was applied at intervals of 1 min (14 stimulations), 5 min (three stimulations), and 10 min (five stimulations) to allow for NE signal recovery.

For fast‐scan cyclic voltammetry recordings (FSCV) of NE in vitro and kinetic analysis and FSCV of in vivo oxygen concentration, see Supplementary Information.

### 2.3 Microdialysis measurements of extracellular NE concentration

Extracellular NE concentration was measured using PFC microdialysis. Mice were anesthetized with a single i.p. injection of urethane (1.5 g/kg) and secured in a stereotaxic frame. A hole was drilled to facilitate the insertion of a microdialysis probe (1 mm membrane length, 0.24 mm diameter, Cuprophane, 6 kDa cut‐off, CMA‐7, Harvard Apparatus, Holliston, MA) into the PFC. The stereotaxic coordinates for the implantation were the same as those used for the insertion of the working electrode in voltammetric recordings (AP: 1.8 mm, ML: 0.25 mm, DV:‐2.0 mm). Following the surgery, the dialysis probe was connected to a CMA 402 syringe pump (Harvard Apparatus) and perfused with aCSF (in mM): 150 Na^+^, 3.0 K^+^, 1.4 Ca^2+^, 0.8 Mg^3+^, 1.0 PO_4_^3−^, and 155 CL^−^ (Harvard Apparatus). We collected 5 samples per mouse following the perfusion rate and time scheme: (1) 5 μL/min for 10 min, (2) 1 μL/min for 1 h, (3) at 1 μL/min for 20 min, (4) 0.2 μL/min for 100 min, and (5) 0.2 μL/min for 100 min. A volume of 5 μL of 4% acetic acid was added to the 20 μL perfusate. The microdialysis samples were then assayed for NE using an Amuza HPLC‐ECD system (San Diego, CA).

### 2.4 PFC NE content

For NE content analysis, the PFC was microdissected and immediately flash‐frozen in liquid nitrogen. The cryovial containing the PFC was stored at −80°C. Samples were homogenized manually in 0.1 M perchloric acid (HClO_4_), centrifuged at 12,000 rpm at 4°C for 5 min, and the supernatant was filtered through 0.22 μm filters. This supernatant was then used for NE determination using HPLC‐ECD and for protein concentration assessment using the BCA method (Pierce BCA Protein Assay Kit, ThermoFisher Scientific, Inc., Rockford, IL). The monoamines were separated on a reverse‐phase column (EICOMPAK CA‐SODS 2.1 mm ID x 150 mm) using a mobile phase consisting of 95%, 0.1 M sodium phosphate buffer with pH 6.0, 400 mg/L sodium octansufonate, 50 mg/L EDTA and 5% methanol at a flow rate of 230 μL/min and detected graphite electrode WE‐3G (EiCOM) set at +450 mV.

### 2.5 Pontine slice preparation for patch‐clamp recording

Brainstem slices (250 μm) containing the LC were prepared from young adult and old C57BL/6 mice. Following deep isoflurane anesthesia, the mouse underwent cardiac perfusion with cold saline solution. The brain and brainstem were swiftly and meticulously dissected and immersed in ice‐cold sucrose‐artificial cerebrospinal fluid (aCSF) containing (in mM): 26 NaHCO_3_, 1.2 NaH_2_PO_4_, 10 glucose, 200 sucrose, 4.5 KCl, 1 MgCl_2_, and 0.5 CaCl_2_. The solution had a pH of 7.4 and osmolarity of 315 mOsm/kg. The pons was sectioned coronally using a vibrating tissue slicer (Leica VT1200S, Wetzlar, Germany). For recovery and holding, slices were kept at room temperature for 1–2 h in aCSF containing (in mM): 25 NaHCO_3_, 1.25 NaH_2_PO_4_, 10 glucose, 124 NaCl, 2.5 KCl, 5 Na‐ascorbate, and 3 Na‐pyruvate. The pH was maintained at 7.4 and the solution was continually aerated with a 95% O2 and 5% CO2 mixture, maintaining an osmolarity of 320 mOsm/kg. Typically, the LC was present in two slices. The slices containing LC were transferred to the recording chamber placed on the stage of a BX51WI Olympus (Tokyo, Japan) microscope. Slices were bathed in aCSF containing (in mM): 25 NaHCO_3_, 1.25 NaH_2_PO_4_, 10 glucose, 124 NaCl, 2.5 KCl, 1 MgCl2, and 2 CaCl_2_. The pH was held at 7.4 and the solution was bubbled with a mixture of 95% O_2_ and 5% CO_2_ to achieve an osmolarity of 320 mOsm/kg. The slices were superfused at a rate of 3–4 mL/min.

### 2.6 Whole‐cell patch‐clamp recording

LC neuron whole‐cell recordings were performed as previously described. (Lee et al., 2024) High‐resistance seals (>2 GΩ) were created for whole‐cell patch‐clamp recordings of LC neurons, utilizing the IR‐DIC technique. Borosilicate glass patch electrodes with filament (Sutter Instruments, Novato, CA) were prepared using a DMZ Zeitz‐puller (Munich, Germany), with resistances ranging from 3 to 6 MΩ. The internal solution used to fill the patch electrodes contained (in mM): 135 K‐gluconate, 5 KCl, 10 HEPES, 0.2 EGTA, 4 Mg‐ATP, and 0.3 Na2‐GTP‐Na. The pH was adjusted to 7.3 with KOH, with an osmolarity of 300 mOsm L^−1^. Current‐clamp recordings were conducted using a Multiclamp 700B amplifier and Clampex 10.7 software (Molecular Devices, San Jose, CA). The electrode capacitance was compensated, and any data with >20% increase in series resistance during recording was discarded. Signals were filtered at 1–4 kHz and digitized at 10–20 kHz with a Digidata 1440 (Molecular Devices). Data analysis was performed using Clampfit 11.2 (Molecular Devices), SigmaPlot 14.5 (Alfasoft, Gothenburg, Sweden), and Excel 2016 (Microsoft, Redmond, WA).

### 2.7 Western blot and protein expression analysis

A western blot analysis was performed to examine Norepinephrine Transporter (NET) and α2a adrenergic receptor protein expression. Protein samples with Laemmli Sample Buffer were boiled in a water bath at 100°C for 4 min and then frozen in a − 20°C freezer until all samples were processed and ready for loading into wells.

Protein samples were loaded onto a CriterionTM TGXTM 26 WELL 8%–16% Precast Gel (BioRad, Hercules, CA) and separated through gel electrophoresis in a denaturing environment using 1x TBS‐SDS Running Buffer (BioRad, Hercules, CA). After electrophoresis, proteins were transferred to a PVDF membrane and subsequently blocked with a 5% non‐fat dry milk solution (NFDMS) in 1x TBST (20 mM Tris, pH 7.5, 500 mM NaCl, 1% Tween) for 1 h at room temperature on a tube rotisserie.

Membranes were then probed with primary antibodies specific to the proteins of interest: rabbit polyclonal anti‐α2 adrenergic receptor (Thermo, Rockford, IL), rabbit polyclonal anti‐NET (Sigma, St. Louis, MO), and mouse monoclonal anti‐β‐Actin (Santa Cruz Biotech, Santa Cruz, CA) in 5% NFDMS. The membranes were incubated with the primary antibodies overnight at 4°C on a tube rotisserie.

The following day, membranes were washed three times for 8 min each with 1x TBST at room temperature, then treated with an HRP‐conjugated secondary antibody: goat anti‐rabbit IgG (H + L) (Thermo, Rockford, IL) or goat anti‐mouse m‐IgG1 BP‐HRP (Santa Cruz Biotech, Santa Cruz, CA) in a 5% NFDMS milk solution. Membranes were incubated with the secondary antibody on the tube rotisserie for 1 h at room temperature, followed by three washes for 8 min each in 1x TBST.

For detection, membranes were covered in ECL Detection Solution. Membrane films were then read using the BioRad ChemiDocTM Imaging System. The initial preview image's strength was used to adjust saturation for better band visualization, typically at a resolution/sensitivity of 4 × 4.

To evaluate β‐actin presence for housekeeping standardization, membranes probed for α2 adrenergic receptor and NET were stripped and reprobed. Membranes were rinsed with TBST, submerged in RestoreTM PLUS Western Blot Stripping Buffer (Thermo, Rockford, IL) for 10 min on a plate shaker, blocked with 5% NFDMS for 1 h at room temperature, and then incubated with anti‐β‐Actin primary and secondary antibodies as previously described.

Images were imported into BioRad Image Lab Software Version 6.1 from the BioRad ChemiDocTM for in‐depth band analysis. Images were inverted for better visualization, and any lane or band misalignment was corrected by rotating the image. Brightness and contrast were adjusted, and lane boxes were outlined equally around all lanes. The “Add Band” option was used to automatically detect the band of interest, which was then reviewed for accuracy. Lane data was exported to Excel, where the Adjusted Band Volume, having the background signal removed from the Total Band Volume, was used for data and graphical analysis.

For methods on gait analysis, net hanging, treadmill, Morris water maze, and novel object recognition, see Supplementary Information.

### 2.8 Data analysis

Data were analyzed using GraphPad Prism software (Version 10, GraphPad Software, San Diego, CA) or SigmaPlot 14.5 (Alfasoft, Gothenburg, Sweeden). NE release data were subjected to two‐way ANOVAs, and when appropriate, Dunnett's multiple comparisons tests were conducted for post‐hoc analysis. In cases of unequal variances between groups, data were analyzed as a percentage of the baseline using nonparametric Friedman tests, where suitable. Data are presented as mean ± SEM, with the level of statistical significance set at *p* < 0.05.

## 3 RESULTS

### 3.1 Cognitive and physical performance deficits in aged mice

**FIGURE 1:**
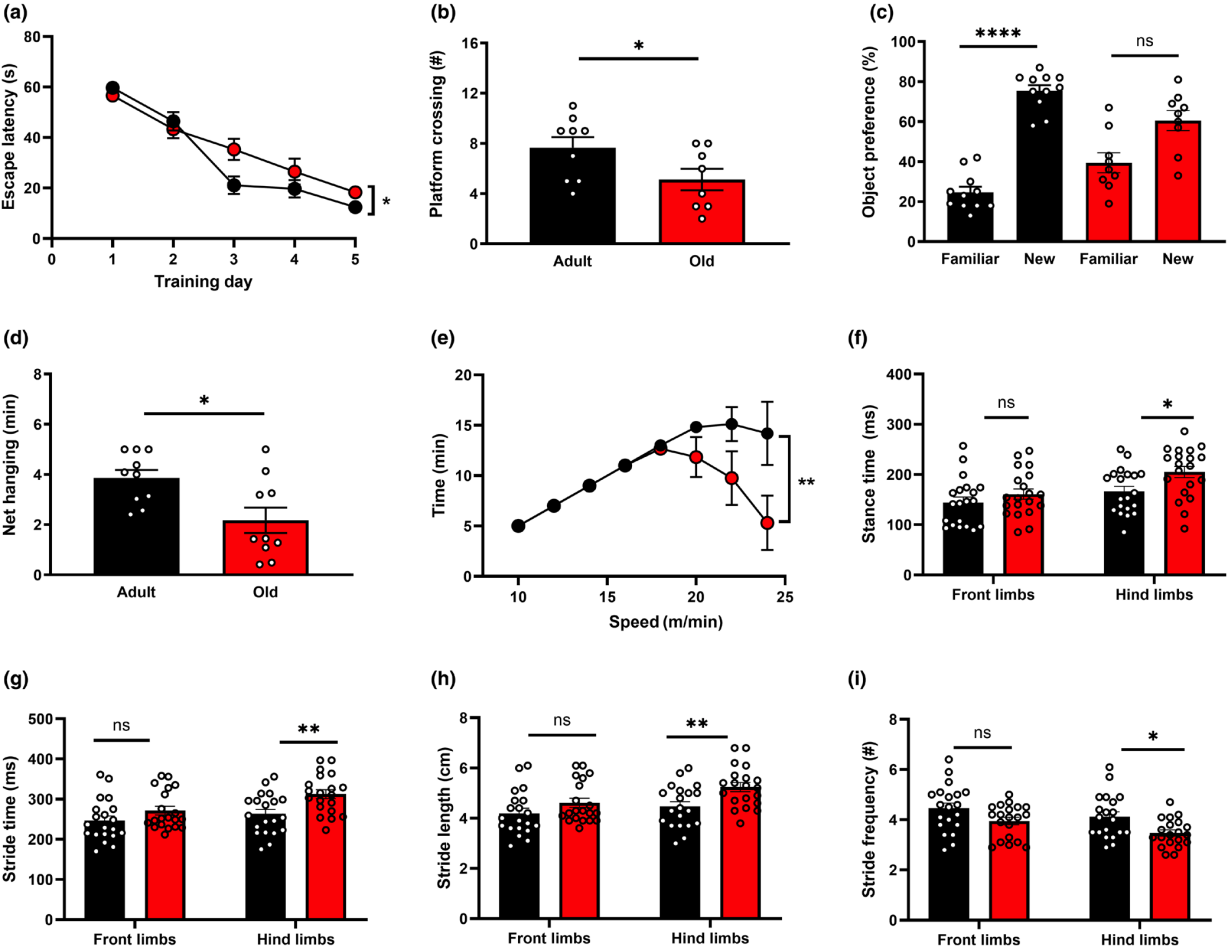
Decline in Cognitive Function and Physical Performance with Aging. (a) The performance of aged mice in the Morris Water Maze, indicated by longer escape latency during the training period is shown in adult control mice (3–5 months) represented by black symbols and old mice (21–23 months) represented by red symbols. (b) Following the probe trial, there were fewer platform crossings observed in old mice (*n* = 8) compared to adult mice (*n* = 9) (*p* = 0.05). (c) The novel object recognition test revealed working memory impairment in aged mice. Adult mice spent significantly more time exploring a new object, whereas there was no significant difference in the time spent by old mice with a familiar and new object (*n* = 9–11 mice per group). (d) Older mice displayed diminished overall body strength compared to adult mice in the net hanging test (*n* = 10 mice per group). (e) Treadmill performance tests indicated lower endurance levels in aged mice compared to their adult counterparts (*n* = 10 mice per group). Significant changes were observed in the gait parameters of old mice, including an increase in hind limb stance time (f), a decrease in stride frequency (g), an increase in stride length (h), and stride time (i) compared to adult mice. (*n* = 9–10 mice per group). Statistical comparisons were made by using two‐way ANOVA (a, e), two‐tailed Student's *t*‐test for unpaired (b,f,g,h,i) and paired (c) data. *p < 0.05, **p < 0.01, ****p < 0.0001; ns indicates non‐significant results (*p* > 0.05).

To compare cognitive and physical performance between old and young‐adult mice, we used established and validated technical approaches. We evaluated cognitive functioning using the Morris Water Maze test and the Novel Object Recognition test. We observed a significant decrease in cognitive performance in aged (21–23 months) mice compared to adult (3–5 months) mice. The results from the Morris Water Maze test revealed that aged mice took significantly longer to locate the hidden platform during the training period, indicative of their poorer spatial learning capabilities (Figure 1a). During the probe trial, aged mice exhibited fewer platform crossings, indicating an impairment in their long‐term memory regarding the platform's location (b). Working memory was further evaluated using the Novel Object Recognition, also known as the Object Preference test. Unlike adult mice who spent a significant amount of time exploring a new object, there was no significant difference observed in the time spent by aged mice between the familiar and new objects, indicating impairment in their working memory (c).

Physical performance was evaluated through the net hanging test, analysis of gait metrics, and a treadmill endurance test. Aged mice exhibited diminished overall body strength, as indicated by their weaker performance in the net hanging test compared to adult mice (d). Furthermore, aged mice displayed reduced endurance levels in the treadmill performance test (e). Lastly, the analysis of gait parameters unveiled notable changes in the locomotor behavior of aging mice. Specifically, older mice demonstrated an increased stance time (f), stride time (g), stride length (h), and front limbs braking time (see Table S1), along with a decreased stride frequency (i). This indicates that to maintain the same speed on the treadmill (17 cm/s), older mice adjusted their gait such that their hind limbs had longer contact (stance) with the belt and moved asynchronously to their front limbs at a slower frequency. Other gait parameters, as detailed in Table S1, did not show significant differences between the two age groups. Taken together, these findings underscore the significant cognitive and physical deficits associated with aging in mice.

### 3.2 Age‐dependent modifications in NE dynamics within the LC‐PFC circuitry

**FIGURE 2:**
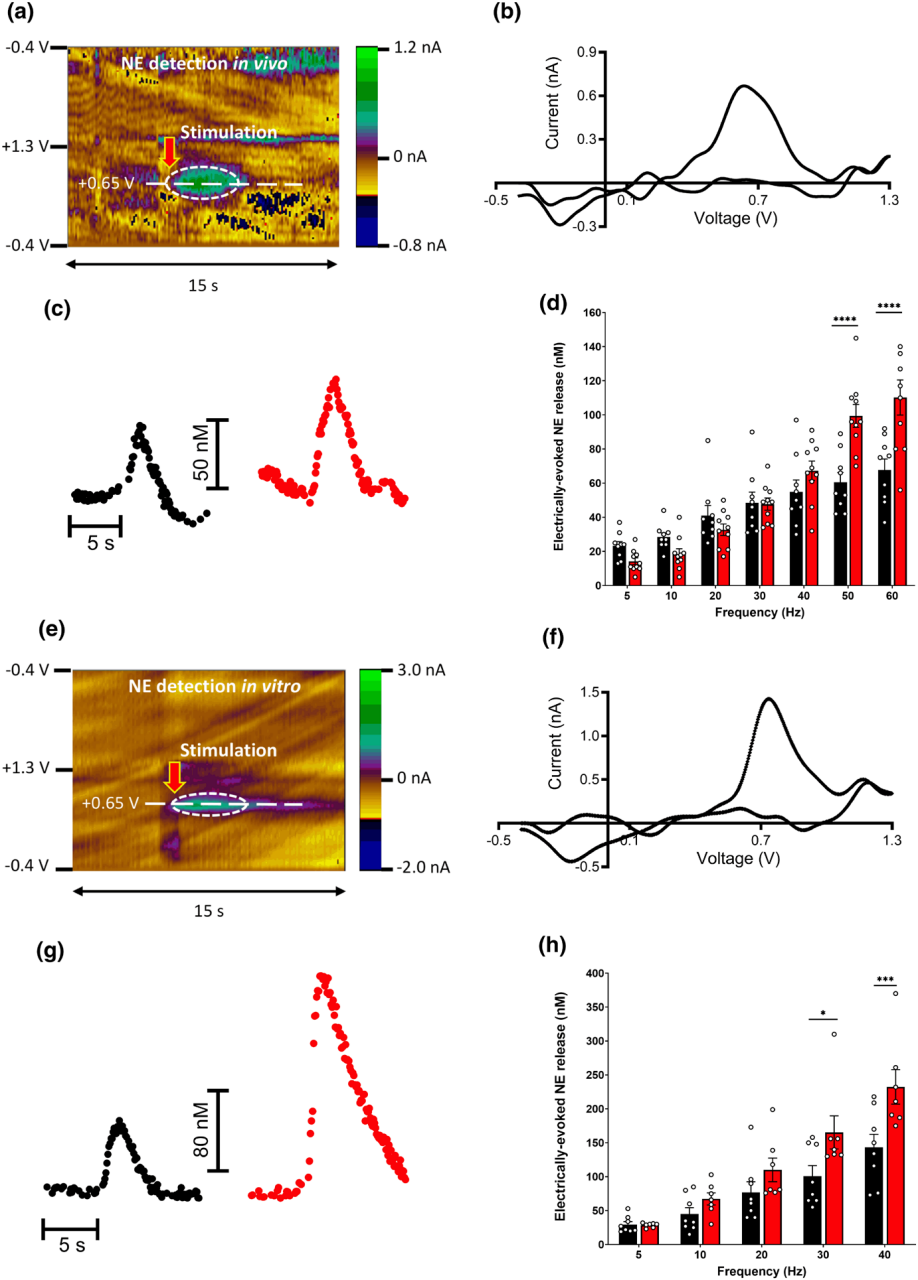
Age‐related Alterations in NE Dynamics within Locus Coeruleus‐Prefrontal Cortex Circuitry. FSCV in conjunction with electrical stimulation was employed to detect NE release in the mouse brain both in vivo (a–d) and in vitro (e–h). (a) The 2D color plots represent the results of electrochemical NE detection in the PFC of an anesthetized mouse. Here, the x‐axis indicates time, the y‐axis shows applied scan potential, and the z‐axis in pseudo‐color represents background‐subtracted faradaic current. The red arrow marks the onset of electrical stimulation. The released substance is oxidized (green spot inside of dashed circle) at a potential optimal for catecholamines. (b) The cyclic voltammogram depicts current measured across the full range of applied potentials at the peak of the detected efflux (center of the green spot on color plots). The color plots and voltammogram suggest the analyte has features (oxidation potential at +0.65 V and reduction potential at −0.2 V) characteristic of catecholamines (NE and dopamine), while pharmacological analysis (Figure S1) verifies that the measured signal is exclusively NE. (c) Representative traces from adult (left) and old (right) anesthetized mice display NE concentration changes following electrical stimulation (50 Hz, 50 pulses, 300 μA) of the LC. (d) Electrical stimulation prompts cortical NE release in a frequency‐dependent manner. The magnitudes of NE release induced by 50 Hz, 1 s—and 60 Hz, 1 s—stimulations were significantly higher in old mice compared to adult controls (mean ± s.e.m., *n* = 9–10 mice per group). (e) The 2D color plots present electrochemical recordings results from the mouse LC slice (see panel a for details). (f) The cyclic voltammogram shows current measured across the full range of applied potentials at the peak of the detected efflux, confirming that the measured substance is NE. (g) Representative traces from adult (left) and old (right) mice display changes in NE concentration in the LC following local electrical stimulation (30 Hz, 30 pulses, 300 μA). (h) Averaged NE responses indicate that higher frequency stimulations resulted in increased effluxes compared to lower frequency stimulations. The magnitudes of NE release induced by 30 Hz, 1 s—and 40 Hz, 1 s—stimulations were significantly higher in old mice compared to adult controls (mean ± s.e.m., *n* = 7–8 mice per group). Statistical comparisons were conducted using two‐way ANOVA with Sidak's multiple comparisons test. P values: *p < 0.05, ***p < 0.01, and ****p < 0.0001.

To detect NE release in the mouse PFC, we utilized fast‐scan cyclic voltammetry (FSCV) along with electrical stimulation. By applying a triangular waveform (ranging from −0.4 V to +1.3 V and back to −0.4 V) to a carbon fiber microelectrode at a frequency of 10 Hz, we were able to oxidize and reduce the examined substance both in vivo (Figure 2a–d) and in vitro (e–h). The results from the electrochemical detection of NE in the PFC of an anesthetized mouse are graphically represented as 2D color plots in Figure 2a. Time is plotted on the *x*‐axis, the applied scan potential on the *y*‐axis, and the background‐subtracted faradaic current is presented on the z‐axis in pseudo‐color. The onset of electrical stimulation is denoted by a red arrow, and the substance released is oxidized at the potential optimal for catecholamines, as highlighted by the green spot within the dashed circle. Figure 2b displays the cyclic voltammogram, which shows the current measured across a range of applied potentials at the peak of the detected efflux. The color plots and voltammogram confirm that the analyte exhibits characteristics typical of catecholamines, specifically an oxidation potential at +0.65 V and a reduction potential at −0.2 V. The pharmacological analysis provided in Figure S2 verifies that the measured signal is uniquely NE.

Representative traces recorded from both adult and old anesthetized mice demonstrate alterations in PFC NE concentration post electrical stimulation (50 Hz, 50 pulses, 300 μA) of the LC (Figure 2c). Interestingly, we observed that electrical stimulation triggers a frequency‐dependent release of NE in the cortex. The magnitudes of electrically‐evoked NE release in response to 50 Hz, 1 s—and 60 Hz, 1 s—stimulations were significantly amplified in old mice compared to adult controls (Figure 2d). Under normal, resting conditions, the firing rate of LC neurons is typically slow and tonic, approximately within the range of 1–3 Hz. However, in instances of heightened arousal or stress, there can be a substantial increase in this firing rate. Therefore, the average spike frequency of LC neurons is not static, but rather it fluctuates according to the physiological or behavioral state of the organism. Patch‐clamp recordings of LC neuron activity demonstrate that the application of positive electrical stimulation can escalate spike frequency across a wide range, (Lee et al., 2024) reaching up to 60 Hz. An illustration for this high‐frequency response is included in Figure S3. Consequently, this provides a reasonable justification for our experimental approach of assessing NE release across the wide range of frequencies that were utilized in Figure 2d.

We further performed electrochemical recordings on mouse LC slices, with results displayed in 2D color plots (Figure 2e). The cyclic voltammogram in Figure 2f reveals the current measured at a range of applied potentials at the peak of the detected efflux, confirming the measured substance as NE. Figure 2g presents representative traces from adult and old mice, showing shifts in NE concentration in the LC following local electrical stimulation (30 Hz, 30 pulses, 300 μA). Upon averaging NE responses, we found that higher frequency stimulations led to heightened effluxes relative to lower frequency stimulations. The electrically‐evoked NE release in response to 30 Hz, 1 s—and 40 Hz, 1 s—stimulations was significantly stronger in old mice compared to adult controls (Figure 2h). In summary, our results indicated a frequency‐dependent release of NE, with older mice showing increased NE release compared to young‐adult counterparts during specific stimulations.

### 3.3 Aging and its influence on NE dynamics within the LC‐PFC pathway

**FIGURE 3:**
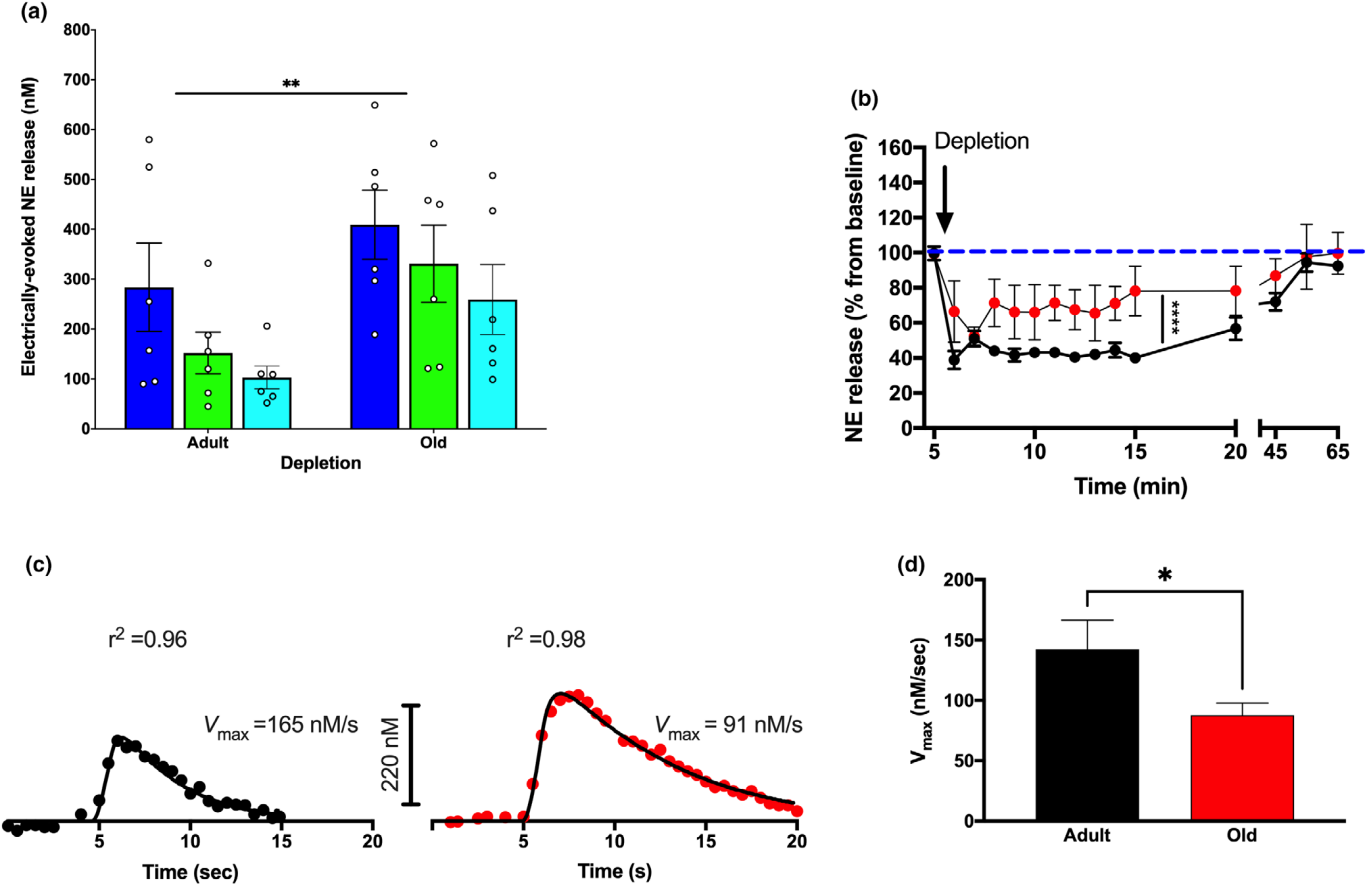
Age‐Induced Changes in the Storage, Recovery and Reuptake of NE within the LC‐PFC Pathway. (a) Old mice displayed greater resistance to NE depletion within the LC/PFC circuit in response to the sequence of stimulations (see the depletion protocol in *Methods*) compared to adult mice. (b) Subsequent to depletion, NE release was allowed to recover (see *Methods*). The outcomes are represented as a percentage of the release observed right before the depleting stimulations. The replenishment trajectories differed significantly between adult (indicated by black symbols and curves) and elderly mice (represented in red). However, both groups took an equivalent duration to completely restore the NE signal (mean ± s.e.m.; *n* = 6 mice per group). (c) Illustration of age‐specific variances in NE reuptake within the mouse LC. Displayed are representative curve fits of electrically‐induced NE variations observed in LC slices from adult (left) and old (right) mice. Each set of data was adjusted to a Michaelis–Menten kinetic model to ascertain the rate of uptake (Vmax). Simulated lines were drafted based on the best alignment with the collected data. For clearer visual representation, individual raw data points are plotted every fifth point. (d) A noteworthy decline in the rate of uptake was observed in old mice in comparison to their adult counterparts (mean ± s.e.m.; *n* = 6–7 mice for each group). For statistical evaluations, a two‐way ANOVA was utilized for parts a and b, while a two‐tailed Student's *t*‐test for independent samples was employed for part d. Significance levels are indicated as: *p < 0.05, **p < 0.01, and ****p < 0.0001.

In our investigations into the NE release patterns within the PFC of anesthetized mice, we noted distinct responses during a series of LC stimulations (Figure 3a). Specifically, older mice demonstrated a remarkable resilience to NE depletion within the LC/PFC circuit upon repeated stimulations, distinguishing them from the adult group. Post depletion, we sought to understand the recovery dynamics of NE release using standard pulse intervals (Figure 3b). While the recovery trajectories were evidently different between adult and elderly cohorts, intriguingly, the time required for a full NE signal restoration remained consistent across both groups.

We next examined variations in NE reuptake associated with aging. Individual data collected in LC slices from each age cohort, were fit to a Michaelis–Menten kinetic model to determine the NE reuptake rate (Figure 3c). This approach revealed a reduced uptake velocity in older mice when compared to their adult counterparts. Of particular note was the evident decline in the uptake rate among the older mice when measured against the adult group (Figure 3d). In summary, age influenced NE depletion and recovery in the LC/PFC pathway, with older mice showing slower NE reuptake rates than young‐adults.

### 3.4 The PFC of aging mice exhibit increased activity and NE content upon stimulation but lower extracellular NE concentration at rest

**FIGURE 4:**
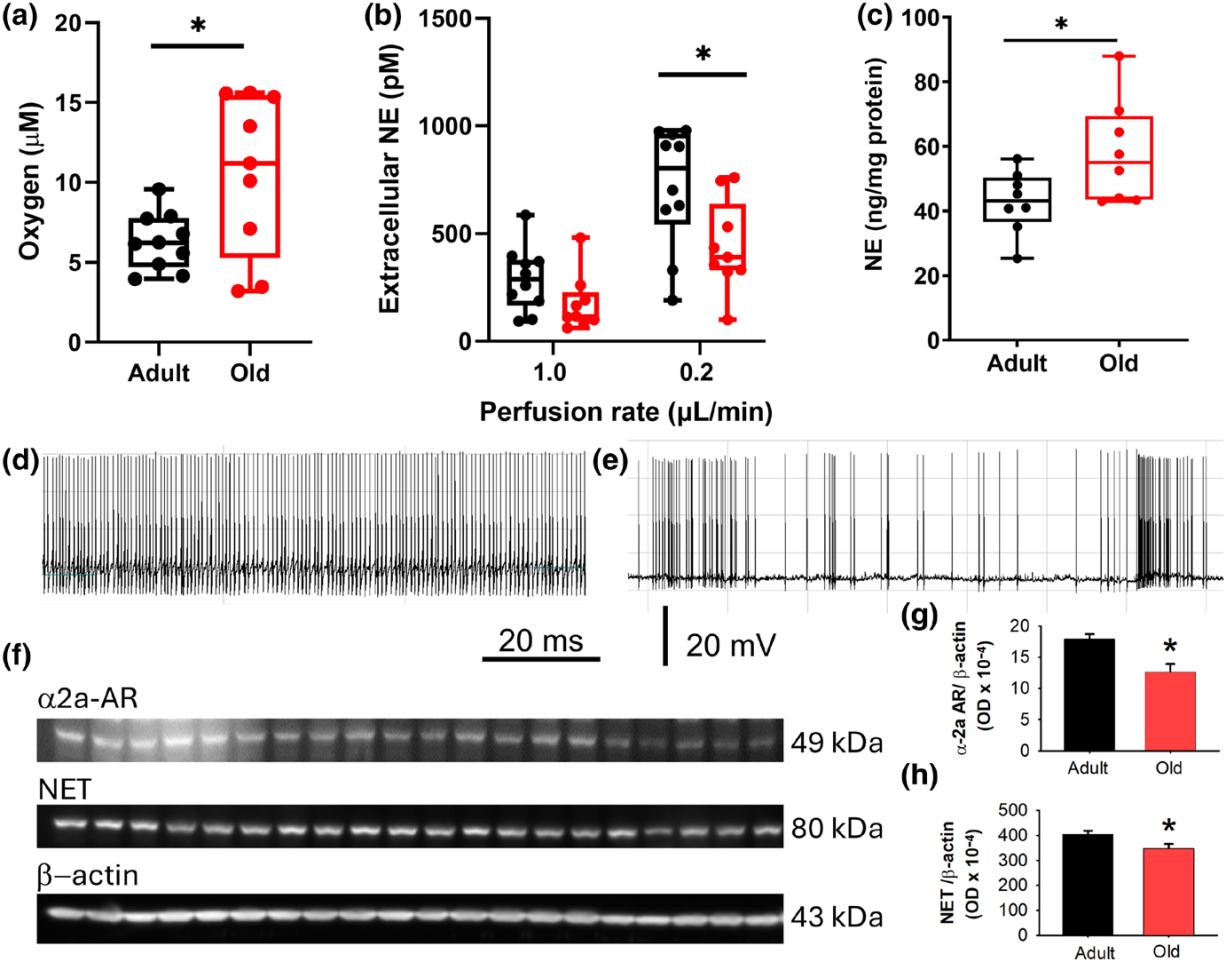
Age‐related changes in norepinephrine dynamics and neuronal activity in the PFC and LC. (a) Oxygen concentration in the PFC upon LC stimulation recorded via voltammetry, showing higher levels in old mice compared to adults (*p < 0.05). (b) Extracellular NE concentration in the PFC as recorded by microdialysis at different perfusion rates. At the lower rate of 0.2 μL/min, a significant difference is observed with old mice showing lower NE levels (*p < 0.05). (c) NE content in the PFC normalized to protein concentration, determined by HPLC, indicating higher NE levels in old mice (*p = 0.026). (d) Trace of spontaneous AP generation in LC neurons from young‐adult mice. (e) Spontaneous action potential generation in LC neurons from an old mice, showing marked spike frequency irregularity. For a detailed analysis of all neurons recorded across the complete mouse cohorts, refer to Tables S2–S4. Calibration bars apply to traces in d and e. (f) PFC Immunoblots for the α2a‐adrenergic receptor, norepinephrine transporter, and β‐Actin were performed using samples from adult male (5), adult female (5), old male (5), and old female (5) mice. The samples were loaded in the same sequence from left to right in the three immunoblots. The protein expression levels of the α2a‐adrenergic receptor (g) and norepinephrine transporter (h) were normalized to β‐Actin and are graphically represented. Statistical significance was denoted by asterisks with *p*‐values of 0.002 and 0.005, respectively.

Figure 4a shows that oxygen levels in the PFC upon LC stimulation are significantly higher in old mice. This suggests increased metabolic and, by extension, heightened neuronal activity in the PFC of older mice compared to adult counterparts. The extracellular NE concentration in the PFC, as determined by microdialysis at two perfusion rates, is significantly greater at the lower rate. In these recording conditions, old mice exhibit lower extracellular NE than young‐adult mice (b), potentially pointing to age‐related changes in baseline NE release.

Analysis of NE content in the PFC, normalized to protein concentration, reveals higher NE levels in old mice (c). This could be a compensatory response to the loss of noradrenergic neurons or altered NE metabolism in aging. The investigation into spontaneous activity and basic membrane properties of individual LC neurons is depicted in (d) and (e) showing traces of spontaneous spike generation from young‐adult and old mice, respectively. Young mice's APs are consistent in frequency, whereas APs in older mice exhibit a significant frequency variation, indicating age‐related variations in ion channel function and/or variations in synaptic input balance. Tables S2,S3 show that neurons from old mice have a more depolarized membrane potential, lower thresholds for activation, and faster kinetics. While cumulative frequency did not differ significantly between age groups, old mice had both higher minimal and maximal frequencies, leading to a more pronounced maximal/minimal frequency ratio.

To evaluate protein expression levels, immunoblots were conducted for the α2a‐adrenergic receptor, NE transporter, and β‐actin using PFC samples from four distinct groups: adult male (5), adult female (5), old male (5), and old female (5) mice (Figure 4f). The protein levels of the α2a‐adrenergic receptor and NE transporter were normalized to β‐actin expression. Quantitative analysis revealed significant differences in protein expression among the age groups. The normalized expression of the α2a‐adrenergic receptor showed a statistically significant age‐dependent decline with a *p*‐value of 0.002 (g). Similarly, the NE transporter also exhibited a significant age‐dependent decline with a *p*‐value of 0.005 (h). These findings indicate notable decrease in the expression of these proteins with aging.

In summary, the study finds that aging is associated with increased neuronal activity in the PFC (as indicated by oxygen levels), altered NE dynamics (with increased total NE content), and facilitation of LC neuron activation. Additionally, significant decreases in the expression of the α2a‐adrenergic receptor and NE transporter were observed, suggesting biochemical alterations in the LC‐PFC circuit with aging. These findings collectively indicate that aging affects both the biochemical and electrical properties of the LC‐PFC circuit, which may underlie observed declines in cognitive and physical performance.

## 4 DISCUSSION

As mammalian species age, their gait often deteriorates more rapidly than cognition. (Longo et al., 2024; Rosano et al., 2004) This slower gait not only serves as an indicator of reduced cognitive function but also strongly predicts a decline in learning and memory abilities associated with the aging process. (Jayakody et al., 2021; Montero‐Odasso et al., 2018) Our research sought to identify significant changes in NE release from the LC and in neuronal activity within the circuit involving the PFC, which is crucial for cognition, planning, posture, and mobility. We focused on mice at an age exhibiting pronounced impairments in learning, reduced muscle strength and endurance, and gait alterations.

### 4.1 An increase in noradrenergic neuron activity in the LC within the PFC is associated with age‐related declines in both cognition and locomotion

Our data reveals that older mice manifest significant declines in learning and spatial memory. This is demonstrated by their reduced exploration of novel objects and increased escape latency, along with fewer platform crossing in the Morris water maze test. This emerging cognitive impairment is observed along with significant motor deterioration, characterized by reduced physical strength and endurance, as well as adjustments in gait including increased stance time, stride time, stride length, and front limbs braking time, but a decrease in stride frequency.

The LC‐PFC pathway is instrumental in modulating cognitive functions. NE release in the PFC has been linked to enhanced performance in tasks involving working memory, attention, and cognitive flexibility. Conversely, a decrease in NE release has been associated with cognitive decline with aging. (Yogev‐Seligmann et al., 2008) Beta‐adrenergic antagonism exacerbates impaired learning and memory mediated by inflammation and pathology in aging rodents. (Evans et al., 2020) While NE is known to be pivotal in higher brain functions, there is less understanding about the effects of elevated NE levels at target areas like the PFC, particularly following activation of its primary source, the LC.

The association between hyperactivity in the LC‐PFC circuit and diminished physical performance in aging has not been previously reported. The interactions between the PFC and other brain regions, suggest a pivotal role for the PFC in gait control, stability and locomotion. Studies have shown that the PFC is involved in planning and initiating gait, controlling gait speed, step length, and frequency. Additionally, the PFC aids in adapting gait to varying environmental conditions, such as obstacles or uneven terrain. The PFC is also believed to play a crucial role in maintaining stability and balance during locomotion. The muscular control of locomotion and gait involves the activation of various muscle groups in a coordinated pattern. (Li et al., 2018) We proposed that brainstem noradrenergic neurons act as a hub at the intersection between cognition, motility and skeletal muscle regulation.(Delbono et al., 2022) NE is a neurotransmitter involved in regulating several functions, including arousal, attention, and stress response. NE neurons located in the brainstem, specifically in the LC, have been shown to play a critical role in modulating gait control. (Takakusaki, 2013) These noradrenergic LC neurons establish synapses with A5 pontine neurons, which in turn project to pre‐ganglionic spinal neurons. (Li et al., 2016) These spinal neurons establish contact with post‐ganglionic noradrenergic neurons located at the paravertebral sympathetic ganglia, supporting an anatomical hierarchy that regulates skeletal muscle innervation, neuromuscular transmission, and muscle trophism. (Delbono et al., 2022).

### 4.2 Impact of enhanced LC‐mediated NE release and neuronal function on the PFC during aging

In this study, we found that, along with increased LC‐mediated PFC NE release, there is also a surge in neuronal activity as measured by oxygen levels through FSCV. It is well‐documented that aging heightens the demands on the PFC during motor control tasks. (Seidler et al., 2010) For instance, older adults demonstrate a higher PFC activation compared to younger adults during regular walking, dual‐task walking, and when avoiding obstacles, indicating an increased cognitive demand during locomotor tasks in older individuals. (Mirelman et al., 2014) In fact, research has shown that older adults with lower gait capacity modulate PFC activation more between fast and slow gait speeds compared to those with higher gait capacity, suggesting a compensatory mechanism involving cognitive processes in older individuals who walk slower. (Harada et al., 2009) These findings strongly suggest that older adults require additional prefrontal activation during locomotion, particularly during dual‐task walking that is cognitively demanding. This might be a compensatory mechanism to engage and leverage additional brain regions that are not as frequently utilized in younger adults, thus enhancing motor task performance. (Cabeza, 2002) This aligns with cognitive resource theories that propose an increased demand in PFC activation during motor control in older adults compared with their younger counterparts, despite the decline in PFC functionality with age. (Seidler et al., 2022) Thus, oxygen levels, serving as a proxy for neuronal function, indicate enhanced neural activity in the PFC of older mice compared to young‐adult mice in response to LC neuron activation.

In summary, hyperactive brainstem noradrenergic neurons can affect gait control and contribute to age‐related gait alterations. The noradrenergic system is a potential target for interventions aimed at improving gait function in older adults.

### 4.3 Mechanisms underlying the age‐related increase in LC‐evoked NE release and neuronal activity in the PFC

Aged mice demonstrated a frequency‐dependent increase in NE release in the PFC in response to LC stimulation, recorded in real‐time, suggesting alterations in neural responsiveness. The relationship between neuron counts in the LC and aging has yielded inconsistent results. (Beardmore et al., 2021) However, a stereological analysis on the LC of C57BL6 mice similar in age to those in our study showed a 20% decrease in NE neurons in older mice (22 months) compared with young‐adult mice (3 months). (Gargano et al., 2020) Downregulation of α2A adrenergic autoreceptors' expression and/or function could potentially compensate for the reduced LC neuron count; yet, the analysis of their levels across various species using different methodologies has produced conflicting results. (Gannon et al., 2015) Our data indicate a decline in α2A adrenergic receptor levels in the PFC with aging, as determined by immunoblot analysis.

Older mice demonstrated resilience to NE depletion in the LC/PFC circuit after repeated stimulations, which aligns with the elevated NE content observed in the PFC. These mice also displayed slower NE reuptake rates compared to adult mice, likely due to decreased NE transporter expression levels. Our data show a decline in NET protein levels in the PFC of our aging mice. This observation correlates with an age‐dependent decline in LC NET availability in healthy aging adults, as determined by positron emission tomography using [^11^C]MRB. (Koohsari et al., 2023) The age‐related downregulation of presynaptic α2A adrenergic receptors may have weakened the negative feedback mechanism, partially accounting for the increased evoked NE efflux post‐depletion. Furthermore, alterations in NE reuptake could potentially contribute to the elevation of prefrontal NE levels.

In aged mice, the PFC displayed reduced extracellular NE levels compared to adults, indicating that, despite decrese NE reuptake, overall levels were lower in older mice. However, the elevated NE content and evoked release in the PFC were associated with a significant increase in neuronal activity, as evidenced by higher oxygen levels in the older group. This finding aligns with observations in humans, where increased neuronal activity has been demonstrated through fMRI studies, (Morcom & Henson, 2018) and our findings that LC neurons in older mice exhibited membrane depolarization, which facilitated their activation.

One potential alternative interpretation is that the increase in NE efflux following depletion might be attributed to age‐related downregulation of presynaptic α2A adrenergic receptors, which are key components of the negative feedback mechanism. Concurrently, the reduced number of NETs could also contribute to the NE elevation, albeit to a lesser extent, by influencing reuptake rates.

The PFC receives NE input from the LC along with dopaminergic input from the ventral tegmental area. Similar to cortical NE, PFC dopamine is a critical modulator of cognitive functions.(Robbins & Arnsten, 2009) Therefore, we cannot exclude the possibility that changes in cortical dopamine may contribute to age‐associated cognitive decline. In fact, impairments in both catecholamines in the PFC have been closely linked to cognitive deficits.(Nelson et al., 2011) It should be noted that dopamine clearance in the PFC is mediated by promiscuous uptake through either the dopamine transporter or the NE transporter.(Carboni et al., 1990) Moreover, dopamine has a higher affinity for the NE transporter than NE itself.(Raiteri et al., 1977) Since the number of NE transporters in the PFC of old mice was significantly decreased, cortical dopamine transmission may be compromised due to an insufficient reuptake rate.

### 4.4 Primary, secondary and compensatory events in the LC‐PFC noradrenergic circuitry with aging

The increased NE content in aged mice could result from compensatory upregulation of NE synthesis in response to the age‐related decrease in extracellular NE concentration. The system may attempt to compensate by producing more NE, leading to an overall increase in tissue NE content. This compensatory response, however, may not fully restore efficient NE signaling, particularly during periods of heightened neuronal activity.

While our study shows increased NE content, it is possible that the dynamics of NE release are altered in aging, leading to more pronounced NE surges during stimulation but a lower baseline release at rest. This could be due to changes in the function of α2A‐adrenergic receptors, which normally inhibit NE release. The observed decline in these receptors might reduce the inhibitory feedback on NE release, contributing to increased NE surges during high‐frequency stimulation, while baseline release remains low due to reduced neuronal activity or other age‐related changes in the release machinery.

The reduced NE uptake rate in aging mice, as observed in our study, suggests that NE clearance from the synapse is impaired. This impaired clearance could lead to prolonged NE signaling at the synapse during stimulation, resulting in higher apparent NE efflux in response to electrical stimulation.

The decline in α2A‐adrenergic receptor expression in the PFC of aged mice could play a significant role in the observed NE dynamics. These receptors are known to inhibit NE release; their reduction might lead to less effective feedback inhibition, thereby amplifying NE release during stimulation.

In conclusion, while the increase in NE content with aging likely contributes to the enhanced NE efflux during stimulation, the altered expression and function of NE transporters and receptors, as well as changes in release dynamics, are also crucial factors that warrant consideration.

Overall, the study reveals a distinctive pattern of NE dynamics in aging, indicating that aging modifies both the structural and functional dynamics within the LC‐PFC neural circuit, affecting NE modulation and neuronal activity. These changes may underlie the observed declines in cognitive and motor functions in aging populations. Understanding these neurochemical and neurophysiological shifts can enhance strategies to mitigate age‐related deficits and improve the quality of life for older adults.

## AUTHOR CONTRIBUTIONS

EB and OD designed the experiments. ZMW, MLM, WRM, JZ, WMS performed the experiments. EB, CM, and OD wrote the manuscript. All authors read and approved the final manuscript.

## FUNDING INFORMATION

The National Institutes of Health grants R01AG057013 and R01AG071545 to Osvaldo Delbono supported this work.

## CONFLICT OF INTEREST STATEMENT

The authors declare that they have no competing interests.

## Supporting information


Data S1.

## Data Availability

All data are available in the main text or the supplementary materials.
