# Supplementary material for: Aging disrupts locus coeruleus‐driven norepinephrine transmission in the prefrontal cortex: Implications for cognitive and motor decline
Source: Aging Cell. 2024 Sep 23;24(1):e14342. doi: 10.1111/acel.14342 (PMC11709105; doi:10.1111/acel.14342)
Supplement: Supplementary file 1 — Data S1. [file ACEL-24-e14342-s001.docx]

**Supporting Information**

**Aging Disrupts Locus Coeruleus-Driven Norepinephrine Transmission in the Prefrontal Cortex: Implications for Cognitive and Motor Decline**

Evgeny Budygin^1^, Valentina Grinevich^1^, Zhong-Min Wang^1^, María Laura Messi^1^,

William Ryan Meeker^1^, Jie Zhang,^2^ William Matthew Stewart,^3^ Carol Milligan^3^,

and Osvaldo Delbono ^1§¥^

**Supplementary Methods**

**Fast-Scan Cyclic Voltammetry Recordings In Vitro**

Mice were anesthetized using isoflurane before decapitation, and their brains were rapidly excised and immediately placed in ice-cold oxygenated artificial cerebrospinal fluid (aCSF) containing: NaCl (126 mM), KCl (2.5 mM), NaH_2_PO4 (monobasic; 1.2 mM), CaCl_2_ (2.4 mM), MgCl_2_ (1.2 mM), L-ascorbic acid (0.4 mM), C_6_H_12_O_6_ (11 mM), and NaHCO_3_ (25 mM). The pH was adjusted to 7.40. Coronal slices (350 µm thick) containing the LC were prepared using a vibrating tissue slicer (Vibratome 1000 Plus, The Vibratome Company, St. Louis, MO, USA), and then placed in oxygenated aCSF at room temperature, allowing 30 minutes for equilibration. Slices were transferred to a submersion recording chamber with room-temperature oxygenated aCSF flowing at a rate of 1 mL/min. The carbon fiber electrode was connected to a voltammetric amplifier (UNC Electronics Design Facility, Chapel Hill, NC, USA) and inserted into the LC (*Figure S1)*. A twisted bipolar stimulating electrode (Plastics One, Roanoke, VA, USA) was positioned on the tissue surface approximately 200 µm from the recording electrode and connected to a voltage output box. NE release was evoked by a single, rectangular pulse 1-second electrical pulse (330 µA, 2 ms/phase, monophasic) at various frequencies (5, 10, 20, 30, and 40 Hz) applied every 10 minutes. Extracellular NE was recorded at the carbon fiber electrode every 100 ms for 15 seconds by applying a triangular waveform (-0.4 V to +1.3 V and back to -0.4 V vs Ag/AgCl, 400 V/s). NE was identified by examining background-subtracted cyclic voltammograms characterized by oxidation and reduction peaks.

***Fast-Scan Cyclic Voltammetry* *Kinetic Analysis***

The rate of extracellular NE uptake, or Vmax, was determined using kinetic modeling (LVIT software, UNC, Chapel Hill, NC, USA) as previously described.(Budygin et al., 1999; Deal, Konstantopoulos, Weiner, & Budygin, 2018; John, Budygin, Mateo, & Jones, 2006) Changes in extracellular NE concentration over time were modeled to follow Michaelis-Menten kinetics. The baseline value of Km was set to 0.480 µM, as determined by single-cell, space-resolved, real-time fluorescence microscopy.(Schwartz, Blakely, & DeFelice, 2003)

***Fast-Scan Cyclic Voltammetry* Recordings *of* *In Vivo Oxygen Concentrations***

Oxygen efflux, evoked electrically, was detected in the PFC of anesthetized mice. Mice were anesthetized using a single i.p. injection of urethane (1.5 g/kg) and positioned securely within a stereotaxic frame. Drilled holes allowed for the insertion of electrodes into the brain. A stimulating electrode (Plastics One) was placed into the LC (AP: -5.3 mm, ML: 0.75 mm DV: -3.2 mm relative to bregma), a carbon fiber working microelectrode was inserted into the ipsilateral PFC (AP: 1.8 mm, ML: 0.25 mm, DV: - 2.0 mm), and an Ag/AgCl reference electrode was implanted into the brain tissue of the contralateral hemisphere. These electrodes were then connected to a voltammetric amplifier, which was interfaced with a computer running specialized software (HDCV, UNC Electronic Facility, Chapel Hill, NC, USA). The pulse for sensing oxygen was an 11 ms waveform that held at 0 V, scanned up to +0.8 V, down to -1.4 V, and then returned to 0 V. (Venton, Michael, & Wightman, 2003; Walton, Boustead, Carroll, & Wightman, 2017; Walton et al., 2021) The signal was confirmed via a background-subtracted cyclic voltammogram, characterized by a reduction peak occurring at approximately -1.3 V. The calibration factor used for calculating oxygen concentrations was -0.19 nA/µM/100 µM.(Walton et al., 2021)

***Protein Extraction***

Protein extraction from the PFC was conducted to evaluate protein levels via Western Blot analysis. The PFC was subjected to RIPA Lysis and Extraction Buffer (25mM Tris-HCl, pH 7.6, 150mM NaCl, 1% NP-40, 1% sodium deoxycholate, 0.1% SDS; Thermo, Rockford, IL), supplemented with a Protease Inhibitor Cocktail (PIC) in DMSO (Sigma, St. Louis, MO). The PIC was added to the RIPA buffer immediately before use to ensure enzyme inhibition. This RIPA-PIC 1% lysis buffer solution was freshly prepared for each extraction. Frozen PFC samples were weighed and the RIPA-PIC 1% lysis buffer was added at a ratio of 10µl per mg of tissue. Samples were placed in 1.5ml Snap Cap Low Retention Microcentrifuge Tubes (Thermo, Rockford, IL) and mechanically homogenized while kept on ice to prevent degradation. Homogenization was performed manually with a plastic pestle, depending on the tissue's consistency. Following thorough homogenization, samples were vortexed to disrupt any remaining tissue fragments and then centrifuged at 10,000g at 4°C for 20 minutes. Total protein concentration was determined using the PierceTM BCA Protein Assay (Pierce, Rockford, IL).

***Gait Analysis***

Gait analysis was carried out using a DigiGait™ Imaging system (Mouse Specifics Inc., Boston, MA, USA) following previously established procedures.(Berryman, Harris, Moalli, & Bagi, 2009) Briefly, a video camera Basler A301fc model (Basler, Inc., Exton, PA) mounted beneath a transparent treadmill belt captures ventral images of the mouse. These images are automatically digitized, and software algorithms analyze them to define the area of each paw. The algorithms generate a set of periodic waveforms that describe the advance and retreat of the four limbs relative to the treadmill belt through consecutive strides. The software identifies the sections of the paw that are in contact with the treadmill belt during the stance and swing phases of the stride and calculates numerous postural and kinematic metrics of gait dynamics. A single mouse was placed on the treadmill, which was set at a speed of 17 cm/sec, a physiological speed at which mice consistently walk for at least four complete strides. Representative values for each parameter were determined by calculating the average of consecutive strides within each 5-second video segment for all four paws. For group comparisons, the averages of the right and left paws were taken to provide representative values for the front and hind paws for each mice.We examined the following 11 indices: (1) *Swing Time* (s), the forward portion of the stride during which the paw is not in contact with the belt; (2) *Stance/Swing* (ratio), the ratio of Stance time to Swing time; (3) *Braking Time* (s), the time between initial paw contact with the belt to the maximal paw contact; (4) *Stance Time* (s), the portion of the stride in which the paw remains in contact with the belt; (5) *% Stance/Stride*, the percentage of time that the stance time contributes to one complete stride cycle; (6) *Stride Length* (cm), the distance between initial contacts of the same paw in one complete stride; (7) *Stride Time* (sec), the amount of time to complete one complete stride for one limb; (8) *% Swing/Stride*, the percent of time that the swing time contributes to one complete Stride cycle; (9) *% Propulsion/Stride*, the percent of time that the propulsion time contributes to one complete stride cycle; (10) *Paw Area* (cm^2^), the maximal paw area in contact with the treadmill during the stance phase of the step cycle; and (11) *Stance Width* (cm), the distance between the two front feet or the two hind feet as measured from the middle of the paw area.(Berryman et al., 2009)

***Net Hanging***

Net hanging, also known as the Inverted-cling grip test, is a method that measures the overall strength and muscular endurance of the mouse. The test was administered using Kondziela's inverted screen test. The mice were allowed to acclimate to the experimental room for 10 minutes before the test commenced, and the duration for which they clung to the net was recorded. The results represent the average of three consecutive trials, as reported in prior research.(Zhang et al., 2016)

***Morris Water Maze***

The Morris Water Maze (MWM), a test used to study spatial memory and learning, was conducted as previously described.(Ma et al., 2013) The training regimen for the hidden platform version of the MWM consisted of four trials per day (with a maximum of 60 seconds per trial and a 15-minute interval between trials) for five consecutive days. A probe trial was conducted two hours after the completion of the training on the fifth day. The visible platform task consisted of four trials per day for two consecutive days. During these trials, the escape platform was marked with a visible cue and was randomly relocated between four locations. The trajectories of the mice during these trials were recorded with a video tracking system (Ethovision XT, Leesburg, VA).

***Novel Object Recognition***

Mice from each age group were tested for their preference towards the objects used during the familiar and novel phases of the experiment. Mouse behavior was recorded with a digital camera (CODi HD 1080P, Garnet Valley, Pennsylvania) and the video recordings were saved and quantified offline. Mice were acclimated to the test room for at least two hours before each session. Low lighting was used to avoid casting any shadows inside the test chamber (47 cm x 47 cm x 47 cm). Objects used were ceramic, glazed shapes (two identical and one novel). Video recordings were started when each animal was placed into the chamber. The chamber and objects were cleaned with 70% ethanol, water, and then dried between each mouse. On day 1 (habituation), animals were placed into the center of the chamber by holding the base of their tail. A timer was started as soon as the tail was released. Mice were allowed to explore the chamber with no objects inside for 15 minutes. On day 2 (familiarization), two identical objects were positioned in the corners of the chamber, and animals were placed into the chamber in the same position as on day 1 for 5 minutes. On day 3 (test day), objects were placed in the same location as on day 2; however, one was replaced with a novel object. Animals were placed into the chamber in the same position as the first two days and allowed to explore the chamber and objects for 5 minutes. The object preference, expressed as a percentage, was calculated by dividing the amount of time spent exploring the novel object by the total time spent exploring both the novel and familiar objects.

***Treadmill***

In-vivo fatigue was recorded using a forced Exer 6 lane treadmill apparatus (Columbus Instruments, Columbus, OH) as described.(Rodrigues et al., 2020) Mice were allowed to run on the treadmill for 5 min at 10 m min^−1^ on days 1 and 2 and 5 min at 10 m min^−1^ followed by 2 m min^−1^ increments until reaching 20 m min^−1^ for 2 min each period on day 3. The treadmill was not inclined. After training, mouse performance was recorded. The initial speed, 10 m min^−1^, reached a maximum of 5 min; we then increased the pace by 2 m min^−1^ every 2 min until exhaustion. We considered the mouse exhausted when it remained at least 10 s in the electric shock area of the treadmill. Maximal tolerated speed was recorded. (Messi, Clark, Prevette, Oppenheim, & Delbono, 2007; Zhang et al., 2016)

**Supplementary Figure Legends**

**Supplementary Figure 1**

**Placement of the Stimulation Electrode in the LC In Vivo and In Vitro Slices. A**. Brightfield image of a control sagittal section from a Dbh-dTomato mouse brainstem and cerebellum, showing no visible damage at the floor of the 4th ventricle, where LC neurons are located (arrow). **B**. Corresponding fluorescence image of the same section, displaying the concentration of noradrenergic neurons in the dorsal pons. **C**. Brightfield image of a sagittal section from a Dbh-dTomato mouse brainstem with an electrode inserted into the LC (indicated by arrow), illustrating pinpoint damage to the dorsal pons. **D**. Corresponding fluorescence image of (**C**), indicating the exact placement of the electrode in the LC. **E**. Brightfield image of a coronal section from a Dbh-dTomato mouse at the pons level, showing the electrode's location during the in vitro experiment. **F**. Corresponding fluorescence image of (**E**), further detailing the electrode's position.

**Supplementary Figure 2**

**Differential Effects of Idazoxan and Raclopride on Catecholamine (CA) Release in the PFC Following LC Electrical Stimulation In Vivo.** Upon establishing a stable baseline of CA efflux, intraperitoneal administration of saline, idazoxan (5 mg/kg), or raclopride (2 mg/kg) was performed as indicated by an arrow. Compared to saline, idazoxan significantly amplified CA release. In contrast, raclopride administr ation did not significantly alter CA efflux. CA efflux data are depicted as a percentage of the average pre-injection baseline for each drug (mean ± SEM). Sample sizes: saline, n=7 mice; idazoxan, n=7 mice; raclopride, n=6 mice. Statistical significance is indicated by *p < 0.05, denoting a significant deviation from the saline control value. A repeated measure two-way ANOVA identified a significant main effect of the drug (F(2,153) = 42.03; p < 0.0001), time (F(8,153) = 4.306; p = 0.0001), as well as a significant interaction (F(16,153) = 4.477; p < 0.0001). Dunnett’s multiple comparison test demonstrated a significant difference (p < 0.01) between the impacts of saline and the α2 adrenergic receptor antagonist (idazoxan) on catecholamine release at the 10, 20, 30, and 50-minute marks following their administration. Conversely, the influence of the D2 dopamine receptor antagonist (raclopride) did not significantly diverge from the saline effect throughout the entire recording period (p > 0.05).

**Supplementary Figure 3**

**High Spike Frequency Induction in LC Neurons.** Positive electrical stimulation for 800-ms increased LC neuron spike frequency to 60Hz. This method was applied in acute pontine slice recordings from Dbh^cre^ KI mice, where noradrenergic neurons were identified by dTomato fluorescence expression for patch-clamp recordings. Panels (**a-b**) display action potentials recordings at half-maximum (a) and maximum (b) frequencies. Panel (**c**) illustrates the relationship between current and spike frequency, with data points derived from traces in (**a**) and (**b**).


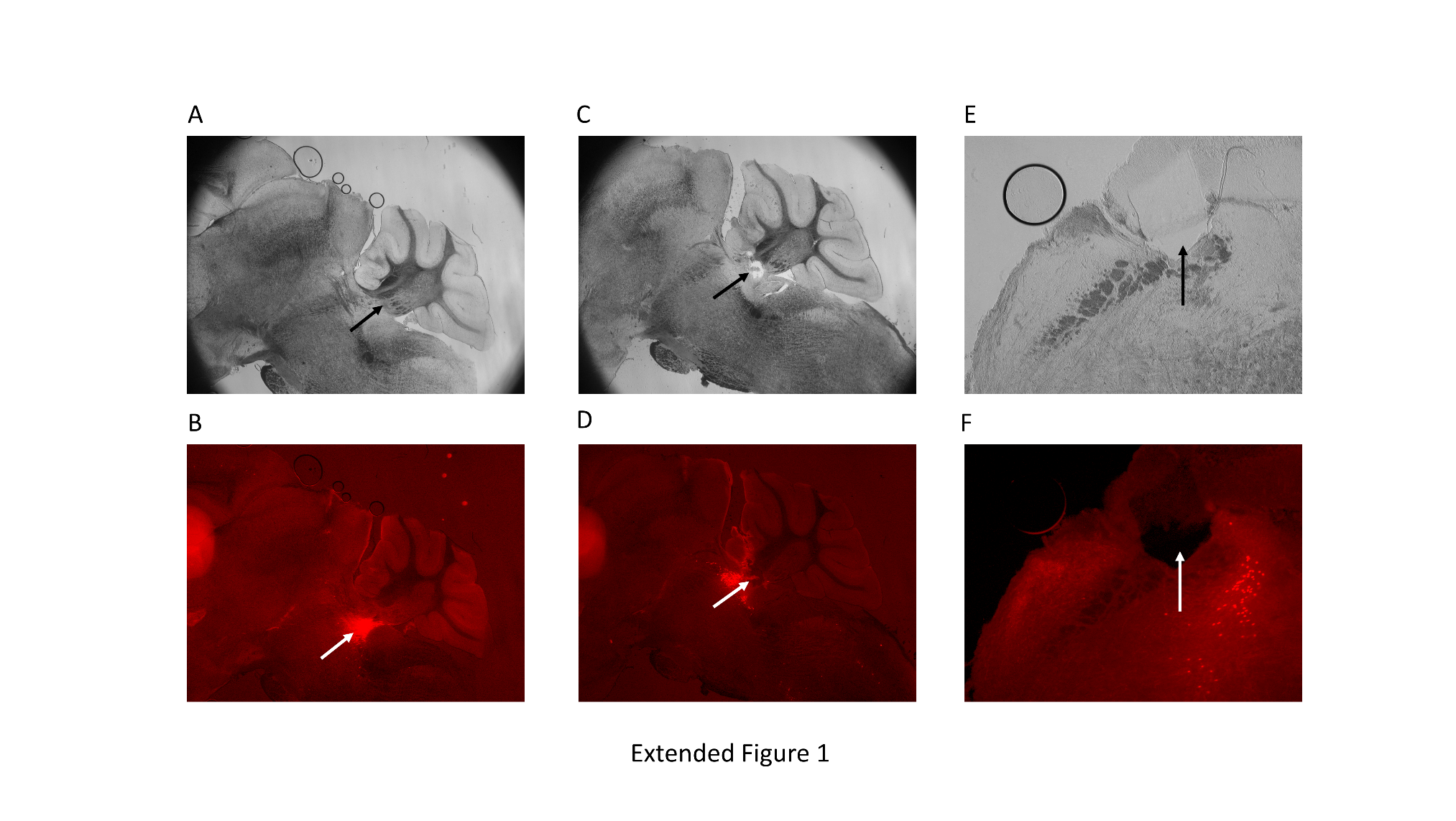


**Supplementary Figure 1**


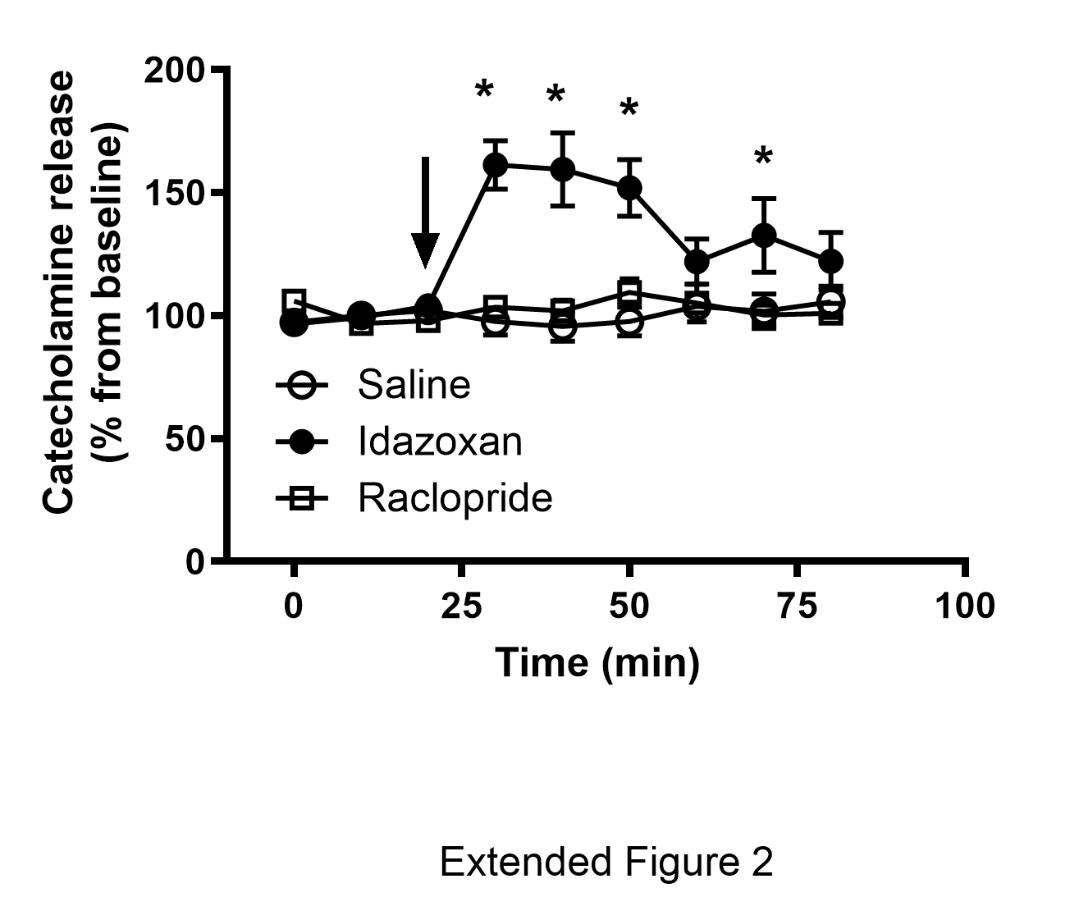


**Supplementary Figure 2**


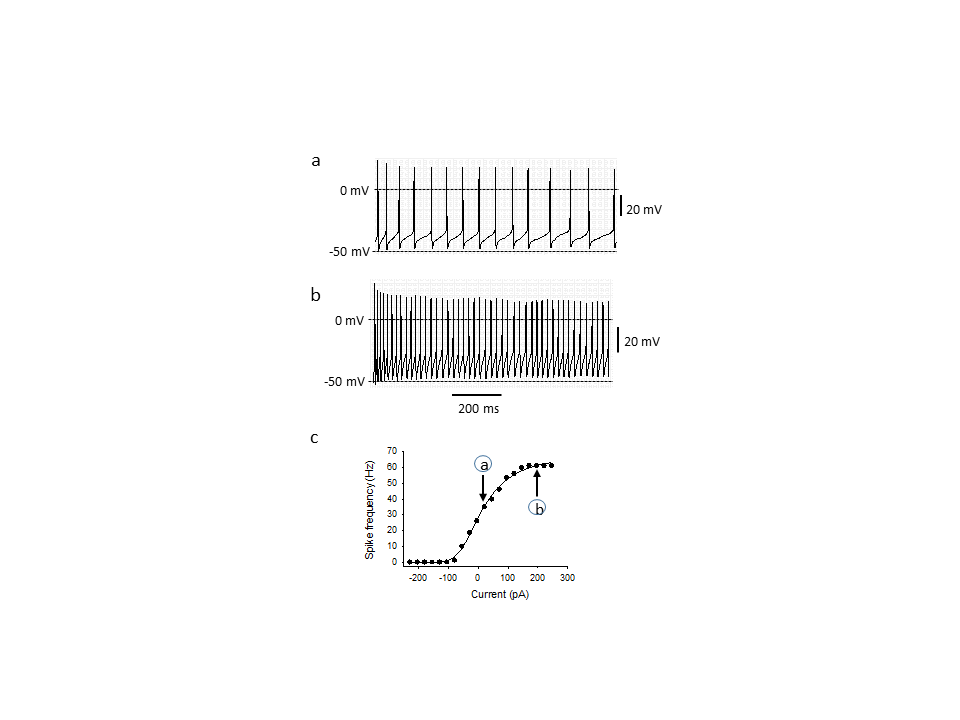


**Supplementary Figure 3**

**Supplementary Table 1**

**Gait Analysis in Adult and Old Mice**

| **Front Limbs** | | | |
| --- | --- | --- | --- |
| **Parameter** | **Adult mice** | **Old mice** | **P value** |
| **Braking time (ms)** | **44.9 ± 2.04** | **60.6 ± 6.19** | **0.024** |
| Propel time (ms) | 90.8 ± 8.14 | 95.0 ± 7.50 | 0.703 |
| Swing time (ms) | 102 ± 5.04 | 110± 8.07 | 0.387 |
| Swing % | 65.1 ± 5.86 | 56.2 ± 4.25 | 0.228 |
| Swing/stride | 42.4 ± 2.13 | 41.0 ± 2.48 | 0.671 |
| Stance width (cm) | 1.80 ± 0.11 | 1.87 ± 0.10 | 0.649 |
| Stance/stride | 57.6 ± 2.13 | 58.9 ± 2.49 | 0.671 |
| Brake/stride | 21.8 ± 1.90 | 24.4 ± 1.76 | 0.319 |
| Brake/stance | 37.9 ± 2.81 | 41.10 ± 2.92 | 0.432 |
| Stance/swing | 1.49 ± 0.14 | 1.59 ± 0.13 | 0.621 |
| Paw area (cm^2^) | 0.16 ± 0.02 | 0.20 ± 0.02 | 0.125 |
| **Hindlimbs** | | | |
| Braking time (ms) | 64.2 ± 4.60 | 83.00 ± 8.29 | 0.056 |
| Propel time (ms) | 102± 10.59 | 122.00 ± 12.42 | 0.236 |
| Swing time (ms) | 96.6 ± 3.76 | 102.70 ± 4.06 | 0.273 |
| Swing % | 48.6 ± 3.89 | 43.1 ± 3.44 | 0.298 |
| Swing/stride | 37.39 ± 1.55 | 34.2 ± 2.00 | 0.218 |
| Stance width (cm) | 2.59 ± 0.10 | 2.82 ± 0.12 | 0.160 |
| Stance/stride | 62.6 ± 1.55 | 65.8 ± 2.00 | 0.218 |
| Brake/stride | 25.2 ± 1.98 | 26.9 ± 2.18 | 0.558 |
| Brake/stance | 37.4 ± 2.54 | 33.6 ± 2.29 | 0.267 |
| Stance/swing | 1.76 ± 0.11 | 2.08 ± 0.15 | 0.079 |
| Paw area (cm^2^) | 0.28 ± 0.03 | 0.38 ± 0.04 | 0.067 |
| **All limbs** | | | |
| Gait Symmetry | 1.09 ± 0.06 | 1.16 ± 0.08 | 0.458 |

|  | Number of Mice | Number of Neurons | Mouse Age  (months) | Sex | Capacitance  (pF) | Input Resistance (MΩ) | Baseline Potential  (mV) |
| --- | --- | --- | --- | --- | --- | --- | --- |
| Young-Adult | 7 | 18 | 2.3 ± 1.3 | 4M, 3F | 65 ± 14 | 475 ± 173 | -50 ± 5.3 |
|  |  |  |  |  |  |  |  |
| Old | 6 | 16 | 23 ± 0.3 | 3M, 3F | 58 ± 18 | 618 ± 246 | -46 ± 5.9 |
|  |  |  |  |  |  |  |  |
| P-value |  |  |  |  | 0.185* | 0.124* | **0.048**** |

**Supplementary Table 2**

**Mice Population and LC Neuron Passive Properties**

Data are expressed as mean ± SD. *Kruskal-Wallis One Way Analysis of Variance on Ranks. **Rank-based ANOVA followed by Dunn’s.

**Supplementary Table 3**

**LC Neuron Spontaneous Action Potential Properties**

|  | Peak AP Amplitude (mV) | Threshold  (mV) | Overshoot  Amplitude  (mV) | AHP  Amplitude  (mV) | AHP  Duration  (ms) | APD at 50%  (ms) | Max Rise Slope  (mV/ms) | Max Decay Slope  (mV/ms) |
| --- | --- | --- | --- | --- | --- | --- | --- | --- |
| Young-Adult | 69 ± 13 | -25 ± 7.2 | 24 ± 7.5 | -12 ± 7.0 | 192 ± 92 | 4.9 ± 1.5 | 33 ± 14 | -18 ± 4.4 |
|  |  |  |  |  |  |  |  |  |
| Old | 71 ± 14 | -30 ± 6.1 | 36 ± 9.3 | -4.9 ± 6.0 | 258 ± 142 | 3.2 ± 0.6 | 65 ± 36 | -27 ± 12 |
|  |  |  |  |  |  |  |  |  |
| P-value | 0.697* | **0.033**** | **0.05**** | **0.003***** | 0.154^#^ | **<0.001***** | **<0.001***** | **0.002***** |

AHP: After hyperpolarization; APD at 50%: Action potential duration at half-amplitude. Data are expressed as mean ± SD. *Kruskal-Wallis One Way Analysis of Variance on Ranks. **Rank-based ANOVA followed by Holm-Sidak. ***Rank-based ANOVA followed by Dunn’s. One-way ANOVA. # One-way ANOVA.

**Supplementary Table 4**

**LC Neuron Spontaneous Action Potentials Frequency Analysis**

|  | Cummulative  Freq. (Hz) | Max Freq (Hz) | Max/Mean  Freq | Min Freq  (Hz) | Max/Min Freq |
| --- | --- | --- | --- | --- | --- |
| Young-Adult | 2.4 ± 1.2 | 4.8 ± 2.3 | 2.4 ± 0.98 | 1.1 ± 0.7 | 5.2 ± 5.5 |
|  |  |  |  |  |  |
| Old | 2.0 ± 1.6 | 8.2 ± 6.4 | 9.2 ± 19 | 0.73 ± 0.96 | 84 ± 193 |
|  |  |  |  |  |  |
| P-value | 0.196* | 0.178* | **0.04**** | **0.047**** | **0.003**** |

Max Freq: Maximal frequency. *Kruskal-Wallis One Way Analysis of Variance on Ranks. **Rank-based ANOVA followed by Dunn’s.

**References**

Berryman, E., Harris, R., Moalli, M., & Bagi, C. (2009). Digigait quantitation of gait dynamics in rat rheumatoid arthritis model. *J Musculoskelet Neuronal Interact, 9*(2), 89-98.

Budygin, E. A., Gainetdinov, R. R., Kilpatrick, M. R., Rayevsky, K. S., Männistö, P. T., & Wightman, R. M. (1999). Effect of tolcapone, a catechol-O-methyltransferase inhibitor, on striatal dopaminergic transmission during blockade of dopamine uptake. *European Journal of Pharmacology, 370*(2), 125-131. doi:<https://doi.org/10.1016/S0014-2999(99)00084-9>

Deal, A. L., Konstantopoulos, J. K., Weiner, J. L., & Budygin, E. A. (2018). Exploring the consequences of social defeat stress and intermittent ethanol drinking on dopamine dynamics in the rat nucleus accumbens. *Scientific reports, 8*(1), 1-9.

John, C. E., Budygin, E. A., Mateo, Y., & Jones, S. R. (2006). Neurochemical characterization of the release and uptake of dopamine in ventral tegmental area and serotonin in substantia nigra of the mouse. *J Neurochem, 96*(1), 267-282. doi:10.1111/j.1471-4159.2005.03557.x

Ma, T., Trinh, M. A., Wexler, A. J., Bourbon, C., Gatti, E., Pierre, P., Cavener, D. R., & Klann, E. (2013). Suppression of eIF2α kinases alleviates Alzheimer's disease-related plasticity and memory deficits. *Nature neuroscience, 16*(9), 1299-1305. doi:10.1038/nn.3486

Messi, M. L., Clark, H. M., Prevette, D. M., Oppenheim, R. W., & Delbono, O. (2007). The lack of effect of specific overexpression of IGF-1 in the central nervous system or skeletal muscle on pathophysiology in the G93A SOD-1 mouse model of ALS. *Exp Neurol, 207*(1), 52-63.

Rodrigues, A. C. Z., Wang, Z.-M., Messi, M. L., Bonilla, H. J., Liu, L., Freeman, W. M., & Delbono, O. (2020). Heart and neural crest derivative 2-induced preservation of sympathetic neurons attenuates sarcopenia with aging. *Journal of Cachexia, Sarcopenia and Muscle, 12*(1), 91-108. doi:<https://doi.org/10.1002/jcsm.12644>

Schwartz, J. W., Blakely, R. D., & DeFelice, L. J. (2003). Binding and transport in norepinephrine transporters: real-time, spatially resolved analysis in single cells using a fluorescent substrate. *Journal of Biological Chemistry, 278*(11), 9768-9777.

Venton, B. J., Michael, D. J., & Wightman, R. M. (2003). Correlation of local changes in extracellular oxygen and pH that accompany dopaminergic terminal activity in the rat caudate–putamen. *Journal of Neurochemistry, 84*(2), 373-381.

Walton, L. R., Boustead, N. G., Carroll, S., & Wightman, R. M. (2017). Effects of glutamate receptor activation on local oxygen changes. *ACS Chemical Neuroscience, 8*(7), 1598-1608.

Walton, L. R., Verber, M., Lee, S.-H., Chao, T.-H. H., Wightman, R. M., & Shih, Y.-Y. I. (2021). Simultaneous fMRI and fast-scan cyclic voltammetry bridges evoked oxygen and neurotransmitter dynamics across spatiotemporal scales. *Neuroimage, 244*, 118634.

Zhang, T., Pereyra, A. S., Wang, Z. M., Birbrair, A., Reisz, J. A., Files, D. C., Purcell, L., Feng, X., Messi, M. L., Feng, H., Chalovich, J., Jin, J. P., Furdui, C., & Delbono, O. (2016). Calpain inhibition rescues troponin T3 fragmentation, increases Cav1.1, and enhances skeletal muscle force in aging sedentary mice. *Aging Cell, 15*(3), 488-498. doi:10.1111/acel.12453
